# Supplementary material for: Prevalence of HER2 overexpression and amplification in cervical cancer: A systematic review and meta-analysis
Source: PLoS One. 2021 Sep 30;16(9):e0257976. doi: 10.1371/journal.pone.0257976 (PMC8483403; doi:10.1371/journal.pone.0257976)
Supplement: S1 File — (DOCX) [file pone.0257976.s001.docx]

**S1 Supplementary file.**

**Bibliographic search**

**Search Strategies**

1. **Embase.**

No. Query Results Results Date

#23. #7 AND #22 603 22 Dec 2020

#22. #8 OR #9 OR #10 OR #11 OR #12 OR #13 OR #14 OR 139,608 22 Dec 2020

#15 OR #16 OR #17 OR #18 OR #19 OR #20 OR #21

#21. (cervix NEAR/1 intraepithelial):ti,ab 20 22 Dec 2020

#20. (cervical NEAR/1 intraepithelial):ti,ab 10,755 22 Dec 2020

#19. 'uterine cervix carcinoma in situ'/exp 15,961 22 Dec 2020

#18. (cervix NEAR/1 adenocarcinom*):ti,ab 237 22 Dec 2020

#17. (cervical NEAR/1 adenocarcinom*):ti,ab 2,025 22 Dec 2020

#16. (cervix NEAR/1 carcinom*):ti,ab 2,598 22 Dec 2020

#15. (cervix NEAR/1 tumor*):ti,ab 151 22 Dec 2020

#14. (cervix NEAR/1 cancer):ti,ab 3,499 22 Dec 2020

#13. (cervix NEAR/1 neoplas*):ti,ab 163 22 Dec 2020

#12. (cervical NEAR/1 carcinom*):ti,ab 14,881 22 Dec 2020

#11. (cervical NEAR/1 tumor*):ti,ab 2,239 22 Dec 2020

#10. (cervical NEAR/1 cancer):ti,ab 65,820 22 Dec 2020

#9. (cervical NEAR/1 neoplas*):ti,ab 3,190 22 Dec 2020

#8. 'uterine cervix tumor'/exp 126,593 22 Dec 2020

#7. #1 OR #2 OR #3 OR #4 OR #5 OR #6 68,633 22 Dec 2020

#6. (neu NEAR/1 receptor*):ti,ab 656 22 Dec 2020

#5. 'her-2':ti,ab 12,898 22 Dec 2020

#4. ('proto oncogene' NEAR/1 protein*):ti,ab 191 22 Dec 2020

#3. cd340:ti,ab 30 22 Dec 2020

#2. 'erbb-2':ti,ab 4,435 22 Dec 2020

#1. 'epidermal growth factor receptor 2'/exp 59,330 22 Dec 2020

**B. Pubmed 20 Dec 2021**

| 23 | #7 AND #22 |  | 185 |
| --- | --- | --- | --- |
| 22 | #8 OR #9 OR #10 OR #11 OR #12 OR #13 OR #14 OR #15 OR #16 OR #17 OR #18 OR #19 OR #20 OR #21 | | 98,341 |
| 21 | Cervix Intraepithelial[tiab] | | 18 |
| 20 | Cervical Intraepithelial[tiab] | | 8,860 |
| 19 | Cervical Intraepithelial Neoplasia[Mesh] | | 10,232 |
| 18 | Cervix Adenocarcinom*[tiab] | | 156 |
| 17 | Cervical Adenocarcinom*[tiab] | | 1,458 |
| 16 | Cervix Carcinom*[tiab] | | 1,424 |
| 15 | Cervix Tumor*[tiab] | | 91 |
| 14 | Cervix Cancer[tiab] | | 2,174 |
| 13 | Cervix Neoplas*[tiab] | | 4,296 |
| 12 | Cervical Carcinom*[tiab] | | 11,743 |
| 11 | Cervical Tumor*[tiab] | | 1,540 |
| 10 | Cervical Cancer[tiab] | | 48,738 |
| 9 | Cervical Neoplas*[tiab] | | 3,120 |
| 8 | Uterine Cervical Neoplasms[Mesh] | | 76,651 |
| 7 | #1 OR #2 OR #3 OR #4 OR #5 OR #6 | | 30,794 |
| 6 | Neu Receptor*[tiab] | | 440 |
| 5 | HER-2[tiab] |  | 7,612 |
| 4 | Proto-Oncogene Protein*[tiab] | | 289 |
| 3 | CD340[tiab] |  | 17 |
| 2 | ErbB-2[tiab] |  | 3,960 |
| 1 | Receptor, ErbB-2[Mesh] | | 25,524 |
|  |  |  |  |

**C. Cochrane Library**

ID Search Hits

#1 MeSH descriptor: [Receptor, ErbB-2] explode all trees 833

#2 ErbB-2:ti,ab,kw with Publication Year to 2020, with Cochrane Library publication date to Dec 2020, in Trials 879

#3 CD340:ti,ab,kw with Cochrane Library publication date to Dec 2020 0

#4 (Proto-Oncogene NEAR/2 Protein*):ti,ab,kw with Cochrane Library publication date to Dec 2020 853

#5 HER-2:ti,ab,kw with Cochrane Library publication date to Dec 2020 2

#6 (Neu NEAR/1 Receptor*):ti,ab,kw with Cochrane Library publication date to Dec 2020 14

#7 #1 OR #2 OR #3 OR #4 OR #5 OR #6 with Cochrane Library publication date to Dec 2020 1725

#8 MeSH descriptor: [Uterine Cervical Neoplasms] explode all trees 2117

#9 (Cervical NEAR/2 Neoplas*):ti,ab,kw with Cochrane Library publication date to Dec 2020 2673

#10 (Cervical NEAR/2 Cancer):ti,ab,kw with Cochrane Library publication date to Dec 2020 3529

#11 (Cervical NEAR/2 Tumor*):ti,ab,kw with Cochrane Library publication date to Dec 2020 65

#12 (Cervical NEAR/2 Carcinom*):ti,ab,kw with Cochrane Library publication date to Dec 2020 539

#13 (Cervix NEAR/2 Neoplas*):ti,ab,kw with Cochrane Library publication date to Dec 2020 94

#14 (Cervix NEAR/2 Cancer):ti,ab,kw with Cochrane Library publication date to Dec 2020 1435

#15 (Cervix NEAR/2 Tumor*):ti,ab,kw with Cochrane Library publication date to Dec 2020 107

#16 (Cervix NEAR/2 Carcinom*):ti,ab,kw with Cochrane Library publication date to Dec 2020 754

#17 (Cervix NEAR/2 Adenocarcinom*):ti,ab,kw with Cochrane Library publication date to Dec 2020 14

#18 (Cervical NEAR/2 Adenocarcinom*):ti,ab,kw with Cochrane Library publication date to Dec 2020 36

#19 MeSH descriptor: [] explode all trees 0

#20 (Cervical NEAR/2 Intraepithelial):ti,ab,kw with Cochrane Library publication date to Dec 2020 1040

#21 (Cervix NEAR/2 Intraepithelial):ti,ab,kw with Cochrane Library publication date to Dec 2020 10

#22 #8 OR #9 OR #10 OR #11 OR #12 OR #13 OR #14 OR #15 OR #16 OR #17 OR #18 OR #19 OR #20 OR #21 with Cochrane Library publication date to Dec 2020 5712

#23 #7 AND #22 with Cochrane Library publication date to Dec 2020 3

**D. Number of records by database**

| EMBASE | 603 |
| --- | --- |
| MEDLINE | 185 |
| COCHRANE | 3 |
| Total | 791 |

**E. Other Sources and Results.**

| **Search date: 22.Dic 2020**  **Source** | **Number of documents retrieved** | **URL** |
| --- | --- | --- |
| Korea Med | 0 | https://www.koreamed.org/ |
| Lilacs | 1 | https://lilacs.bvsalud.org/es/ |
| Bireme | 0 | https://bvsalud.isciii.es/blog/tag/bireme/ |
| Index Medicus for Southeastern Asia Región | 0 | https://onlinelibrary.london.ac.uk/search/node/cervical%20cancer%20AND%20HER2 |
| African index Medicus | 0 | https://indexmedicus.afro.who.int/ |
| Western Pacific region Index Medicus | 0 | https://www.globalindexmedicus.net/biblioteca/wprim/#:~:text=The%20Western%20Pacific%20Region%20Index,of%20the%20Western%20Pacific%20Region. |
| Google Scholar | 1 | https://scholar.google.com/ |
| Open Access Theses and Dissertations | 0 | https://oatd.org/ |
