# Supplementary material for: Prevalence of HER2 overexpression and amplification in cervical cancer: A systematic review and meta-analysis
Source: PLoS One. 2021 Sep 30;16(9):e0257976. doi: 10.1371/journal.pone.0257976 (PMC8483403; doi:10.1371/journal.pone.0257976)
Supplement: S2 File — (DOCX) [file pone.0257976.s002.docx]

**Additional file 2.**

**Table. Data items.**

|  |  | **Paper features** | | | |
| --- | --- | --- | --- | --- | --- |
| Count | SR ID number | ID: Last Name of the first author Year of report | Author | Title | Journal |

| **Location** | | **Data Period** | | **Design** | **N** |
| --- | --- | --- | --- | --- | --- |
| WHO Region | Country | Start (mm/yyyy) | End (mm/yyyy) | Study Design | N |

|  | **Patient characteristics** | | **Tumor** | **characteristics** |
| --- | --- | --- | --- | --- |
| Age (Median) | Age Lower Limit per protocol | Age Upper Limit per protocol | Histology | FIGO/TNM Stage |

| **Sample characteristics** | | | | |
| --- | --- | --- | --- | --- |
| Sampling Method | Sampling Site | Sample storage | Sampling to Fixation Time (days) | Fixation to Assay Time(month) |

| **Assay characteristics IHC** |  |  |  | **Assay characteristics IHC** | |
| --- | --- | --- | --- | --- | --- |
| Method Used | Total N analyzed | Staning platform | Visualization system | Primary Antibody | Positivity Definition |

| **Assay characteristics Hybridization** | |
| --- | --- |
| Probe | Positivity definition |

|  | **Results IHC** | | | |
| --- | --- | --- | --- | --- |
| N Analyzed IHC | N 2+ IHC | % 2 +IHC | N 3+ IHC | % 3+ IHC |

| **Results Hybridization** | | |
| --- | --- | --- |
| N Analyzed Hybridization | N Positive Hybridization | % Positive Hybridization |

| **Total Results** | | | |
| --- | --- | --- | --- |
| Total N Positive | Total % Positive (Prevalence) | Total N Undetermined | Total % Undetermined |

| **HPV positive** |
| --- |
| HPV positive **%** |
