# Supplementary figures and images for: Prevalence of HER2 overexpression and amplification in cervical cancer: A systematic review and meta-analysis
Source: PLoS One. 2021 Sep 30;16(9):e0257976. doi: 10.1371/journal.pone.0257976 (PMC8483403; doi:10.1371/journal.pone.0257976)

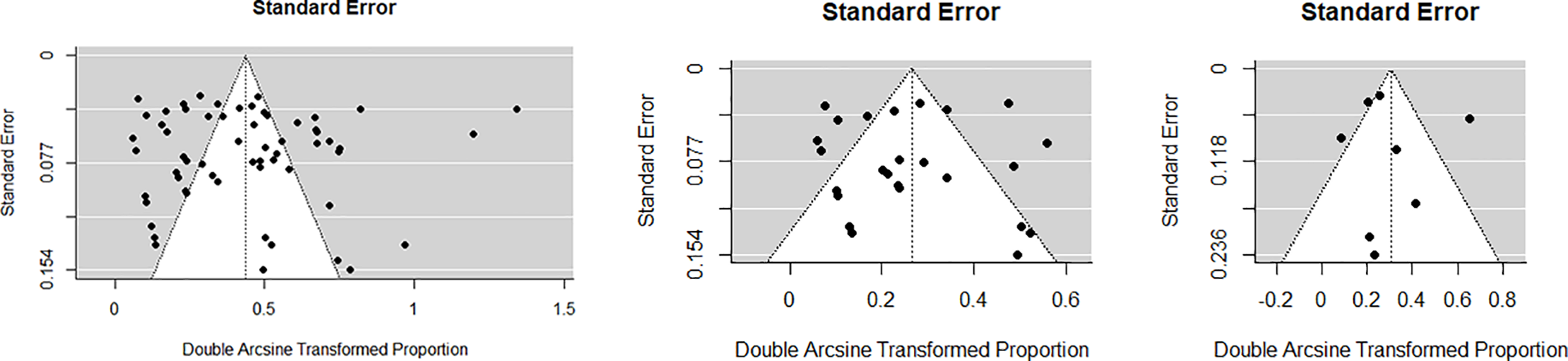

Supplement: S3 File — A Funnel ihc all. B Funnel compliant. C Funnel ish. (TIFF) [file pone.0257976.s003.tiff]

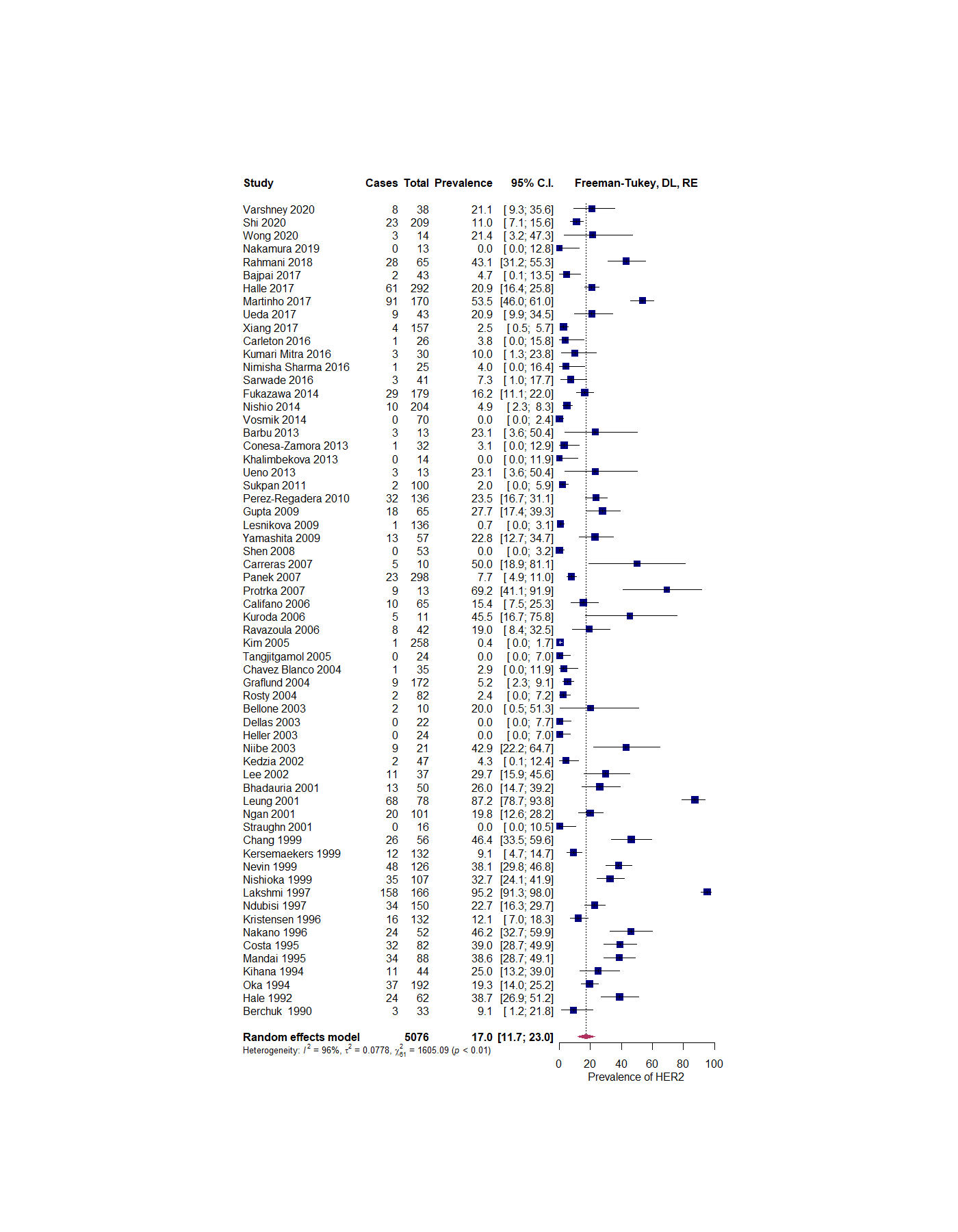

Supplement: S9 File — (TIFF) [file pone.0257976.s009.tiff]
