# Supplementary material for: Prevalence of HER2 overexpression and amplification in cervical cancer: A systematic review and meta-analysis
Source: PLoS One. 2021 Sep 30;16(9):e0257976. doi: 10.1371/journal.pone.0257976 (PMC8483403; doi:10.1371/journal.pone.0257976)
Supplement: S4 File — (DOCX) [file pone.0257976.s004.docx]

**S4 Supplementary file.**

**Table. Study characteristics.**

| Author | Year of publication | Start | End | Study Design |
| --- | --- | --- | --- | --- |
| Varshney | 2020 | NA | NA | case-series |
| Shi | 2020 | 2014 | 2019 | retrospective cohort |
| Wong | 2020 | 1994 | 2019 | retrospective cohort |
| Nakamura | 2019 | 2010 | 2016 | retrospective cohort |
| Rahmani | 2018 | NA | NA | case-series |
| Kumari Mitra | 2018 | NA | NA | case-series |
| Bajpai | 2017 | 2015 | 2016 | cross-sectional |
| Halle | 2017 | 2001 | 2014 | prospective cohort |
| Martinho | 2017 | NA | NA | retrospective cohort |
| Ueda | 2017 | 2004 | 2012 | retrospective cohort |
| Xiang | 2017 | 2010 | 2014 | cross-sectional |
| Carleton | 2016 | NA | NA | case-series |
| Sarwade | 2016 | 2013 | 2015 | case-control |
| Nimisha Sharma | 2016 | NA | NA | prospective and retrospective cohort |
| Fukazawa | 2014 | 1985 | 2001 | retrospective cohort |
| Nishio | 2014 | 1995 | 2007 | retrospective cohort |
| Vosmik | 2014 | 1998 | 2008 | retrospective cohort |
| Barbu | 2013 | 2005 | 2010 | case-series |
| Coneza-Zamora | 2013 | 2002 | 2011 | case-series |
| Khalimbekova | 2013 | 1960 | 2010 | retrospective cohort |
| Ueno | 2013 | 1988 | 2011 | case-series |
| Sukpan | 2011 | 1995 | 2008 | case-series |
| Perez-Regadera | 2010 | 1994 | 2004 | retrospective cohort |
| Gupta | 2009 | NA | NA | case-series |
| Lesnikova | 2009 | NA | NA | cross-sectional |
| Yamashita | 2009 | 1998 | 2005 | retrospective cohort |
| Shen | 2008 | 2002 | 2003 | retrospective cohort |
| Carreras | 2007 | NA | NA | case-series |
| Fuchs | 2007 | 1987 | 1994 | case-series |
| Panek | 2007 | 1985 | 200 | retrospective cohort |
| Protrka | 2007 | 2004 | 2005 | case-control |
| Califano | 2006 | NA | NA | case-series |
| Kuroda | 2006 | 1990 | 2003 | case-series |
| Ravazoula | 2006 | NA | NA | retrospective cohort |
| Kim | 2005 | 1989 | 2001 | retrospective cohort |
| Tangjitgamol | 2005 | 1975 | 1998 | case-series |
| Chavez Blanco | 2004 | NA | NA | case-series |
| Graflund | 2004 | 1965 | 1989 | retrospective cohort |
| Rosty | 2004 | NA | NA | retrospective cohort |
| Bellone | 2003 | 1998 | 2000 | case-series |
| Dellas | 2003 | 1986 | 1995 | case-series |
| Heller | 2003 | 1994 | 2000 | retrospective cohort |
| Niibe | 2003 | 1987 | 1995 | prospective cohort |
| Kedzia | 2002 | 1994 | 1997 | case-series |
| Lee | 2002 | 1993 | 1998 | cross-sectional |
| Bhaduria | 2001 | NA | NA | case-series |
| Leung | 2001 | 1987 | 1994 | cross-sectional |
| Ngan | 2001 | NA | NA | retrospective cohort |
| Straughn | 2001 | 1978 | 1999 | case-series |
| Chang | 1999 | NA | NA | case-series |
| Kersemaekers | 1999 | 1984 | 1995 | case-series |
| Mark | 1999 | NA | NA | case-series |
| Nevin | 1999 | 1982 | 1989 | retrospective cohort |
| Nishioka | 1999 | NA | NA | retrospective cohort |
| Sharma | 1999 | NA | NA | retrospective cohort |
| Lakshmi | 1997 | NA | NA | cross-sectional |
| Ndubisi | 1997 | 1986 | 1992 | retrospective cohort |
| Kristensen | 1996 | 1987 | 1990 | case-series |
| Nakano | 1996 | 1988 | 1990 | prospective cohort |
| Costa | 1995 | 1970 | 1993 | retrospective cohort |
| Mandai | 1995 | 1975 | 1989 | case-control |
| Kihana | 1994 | 1962 | 1989 | case-series |
| Oka | 1994 | 1975 | 1980 | retrospective cohort |
| Hale | 1992 | NA | NA | retrospective cohort |
| Berchuk | 1990 | 1987 | 1989 | case-series |

Abbreviations: NA = information not available.
