# Supplementary material for: Prevalence of HER2 overexpression and amplification in cervical cancer: A systematic review and meta-analysis
Source: PLoS One. 2021 Sep 30;16(9):e0257976. doi: 10.1371/journal.pone.0257976 (PMC8483403; doi:10.1371/journal.pone.0257976)
Supplement: S5 File — (DOCX) [file pone.0257976.s005.docx]

**S5 Supplemental file.**

**Table S5. Geographic information about the included studies.**

| Author | Year | Who region | Country |
| --- | --- | --- | --- |
| Shi | 2020 | Asia Pacific | China |
| Wong | 2020 | Asia Pacific | China |
| Varshney | 2020 | SEA | India |
| Nakamura | 2019 | Asia Pacific | Japan |
| Rahmani | 2018 | Eastern Mediterranean | Sudan |
| Kumari Mitra | 2018 | SEA | India |
| Bajpai | 2017 | SEA | India |
| Halle | 2017 | Europe | Norway |
| Martinho | 2017 | Americas | Brazil |
| Ueda | 2017 | Asia Pacific | Japan |
| Xiang | 2017 | Asia Pacific | China |
| Carleton | 2016 | Americas | UK |
| Sarwade | 2016 | SEA | India |
| Nimisha Sharma | 2016 | SEA | India |
| Fukazawa | 2014 | Americas | Brazil |
| Nishio | 2014 | Asia Pacific | Japan |
| Vosmik | 2014 | Europe | Czech Republic |
| Barbu | 2013 | Europe | Romania |
| Coneza-Zamora | 2013 | Europe | Spain |
| Khalimbekova | 2013 | Europe | Russia |
| Ueno | 2013 | Asia Pacific | Japan |
| Sukpan | 2011 | SEA | Thailand |
| Perez-Regadera | 2010 | Europe | Spain |
| Gupta | 2009 | SEA | India |
| Lesnikova | 2009 | Europe | Denmark |
| Yamashita | 2009 | Asia Pacific | Japan |
| Shen | 2008 | Asia Pacific | China |
| Carreras | 2007 | Europe | Spain |
| Fuchs | 2007 | Europe | Germany |
| Panek | 2007 | Europe | Poland |
| Protrka | 2007 | Europe | Serbia |
| Califano | 2006 | Europe | Italy |
| Kuroda | 2006 | Asia Pacific | Japan |
| Ravazoula | 2006 | Europe | Greece |
| Kim | 2005 | Americas | Korea |
| Tangjitgamol | 2005 | Americas | USA |
| Chavez Blanco | 2004 | Americas | Mexico |
| Graflund | 2004 | Europe | Sweden |
| Rosty | 2004 | Europe | France |
| Bellone | 2003 | Americas | USA |
| Dellas | 2003 | Europe | Switzerland |
| Heller | 2003 | Americas | USA |
| Niibe | 2003 | Asia Pacific | Japan |
| Kedzia | 2002 | Europe | Poland |
| Lee | 2002 | Asia Pacific | Korea |
| Bhaduria | 2001 | SEA | India |
| Leung | 2001 | Asia Pacific | China |
| Ngan | 2001 | Asia Pacific | China |
| Straughn | 2001 | Americas | USA |
| Chang | 1999 | Asia Pacific | China |
| Kersemaekers | 1999 | Europe | Netherlands |
| Mark | 1999 | Americas | USA |
| Nevin | 1999 | Europe | UK |
| Nishioka | 1999 | Europe | UK |
| Sharma | 1999 | SEA | India |
| Lakshmi | 1997 | SEA | India |
| Ndubisi | 1997 | Americas | USA |
| Kristensen | 1996 | Europe | Norway |
| Nakano | 1996 | Asia Pacific | Japan |
| Costa | 1995 | Americas | USA |
| Mandai | 1995 | Asia Pacific | Japan |
| Kihana | 1994 | Americas | Japan |
| Oka | 1994 | Asia Pacific | Japan |
| Hale | 1992 | Europe | UK |
| Berchuk | 1990 | Americas | USA |

Abbreviations: WHO = World Health Organization. SEA = South Eastern Asia. UK = United Kingdom. USA = United States of America.
