# Supplementary material for: Prevalence of HER2 overexpression and amplification in cervical cancer: A systematic review and meta-analysis
Source: PLoS One. 2021 Sep 30;16(9):e0257976. doi: 10.1371/journal.pone.0257976 (PMC8483403; doi:10.1371/journal.pone.0257976)
Supplement: S6 File — (DOCX) [file pone.0257976.s006.docx]

**S6 Supplementary file.**

**Table. Characteristics of In Situ Hybridization methods of the included studies.**

| Study | Year | N patients | ISH type | Dual or single | Ratio for positivity | Average copy number for positivity | Probe |
| --- | --- | --- | --- | --- | --- | --- | --- |
| Shi | 2020 | 209 | FISH | Dual | 2 | ASCO/CAP 2018 | Path-Vision DNA Probe Kit  (Abbott Molecular Inc., Des Plaines,IL, USA) |
| Nakamura | 2019 | 13 | DISH | Dual | 2 | NA | NA |
| Xing | 2017 | 157 | FISH | Dual | NA | NA | Path-Vision DNA Probe Kit |
| Coneza-Zamora | 2013 | 32 | FISH | Dual | 3 | 10 or more in 5% of cells | HER-2/neu/Alphasat. 17 (red/green) cat: RFONC1712 (Qbiogene, Montreal,Canada |
| Ueno | 2013 | 8 | FISH | Dual | 2 | NA | Path-Vysion DNA Probe Kit (Vysis, Downers Grove, IL) |
| Lesnikova | 2009 | 136 | CISH | Single | NA | 5 o more | SPOT-Light HER2 CISH kit (Zymed Inc., SouthSan Francisco, CA, USA) |
| Fuchs | 2007 | NA | FISH | NA | NA | 4 or more | VentanaBenchmark®immunostainer (Ventana Medical Systems, Tucson, Arizona, USA) |
| Chavez Blanco | 2004 | 4 | FISH | Dual | 2 | NA | Path-Vysion DNA Probe Kit (Vysis) |
| Rosty | 2004 | 5 | FISH | Single | NA | 6 or more | Spot-Light HER-2 DNA probe (Zymed Laboratories) |
| Mark | 1999 | 23 | FISH | Dual | 1.5 | NA | LSI HER-2/neu SpectrumOrange/CEP17 SpectrumGreen Dual Color Probe from Vysis(DownersGrove,IL) |
| Sharma | 1999 | 60 | FISH | NA | arbitrary | arbitrary | 3digoxigenin-labelled probe (Oncor Inc., USA) |

Abbreviations: ISH = In Situ Hybridization. NA = information not available
