# Supplementary material for: Prevalence of HER2 overexpression and amplification in cervical cancer: A systematic review and meta-analysis
Source: PLoS One. 2021 Sep 30;16(9):e0257976. doi: 10.1371/journal.pone.0257976 (PMC8483403; doi:10.1371/journal.pone.0257976)
Supplement: S7 File — (DOCX) [file pone.0257976.s007.docx]

**S7 Supplementary file.**

**A. Clinical features of patients in studies included in the review.**

| Study | Publication year | Age, median | Age, the lower limit | Age, the upper limit | FIGO stage | HPV positive, % |
| --- | --- | --- | --- | --- | --- | --- |
| Varshney | 2020 | NA | NA | NA | 1,2,3,4 | NA |
| Shi | 2020 | 49 | 27 | 68 | 1,2,3,4 | 68 |
| Wong | 2020 | 52 | 34 | 86 | 1,2,3,4 | 75 |
| Nakamura | 2019 | 51 | 38 | 72 | 1,2,3,4 | NA |
| Rahmani | 2018 | 65 | 26 | 98 | 1,2,3,4 | NA |
| Kumar Mitra | 2018 | NA | 21 | 80 | 2,3,4 | NA |
| Bajpai | 2017 | NA | NA | NA | NA | NA |
| Halle | 2017 | 42 | NA | NA | 1,2,3,4 | 100 |
| Martinho | 2017 | 47 | 21 | 84 | NA | NA |
| Ueda | 2017 | 43 | 25 | 79 | 1,2,3 | NA |
| Xiang | 2017 | 47 | NA | NA | 1,2 | 55 |
| Carleton | 2016 | NA | NA | NA | NA | NA |
| Sarwade | 2016 | NA | NA | NA | 1,2,3,4 | NA |
| Nimisha Sharma | 2016 | NA | NA | NA | 1,2,3,4 | NA |
| Fukazawa | 2014 | NA | NA | NA | 1 | NA |
| Nishio | 2014 | 49 | 19 | 77 | 1,2 | NA |
| Vosmik | 2014 | 50 | 28 | 81 | 1,2,3,4 | 79 |
| Barbu | 2013 | 56 | 45 | 69 | 1,2,3 | NA |
| Conesa-Zamora | 2013 | NA | NA | NA | NA | 100 |
| Khalimbekova | 2013 | 42 | 10 | 77 | 1,2,3,4 | 0 |
| Ueno | 2013 | 67 | 36 | 85 | 1,2,3,4 | 0 |
| Sukpan | 2011 | 42 | 25 | 80 | 1,2,3,4 | NA |
| Perez Regadera | 2010 | NA | NA | NA | 1,2,3,4 | NA |
| Gupta | 2009 | NA | NA | NA | 1,2,3,4 | NA |
| Lesnikova | 2009 | NA | NA | NA | NA | NA |
| Yamashita | 2009 | 61 | 27 | 82 | 2,3,4 | NA |
| Shen | 2008 | 48 | 29 | 72 | 1,2,3 | NA |
| Carreras | 2007 | NA | NA | NA | NA | 100 |
| Panek | 2007 | 52 | 21 | 79 | 1,2 | NA |
| Protrka | 2007 | NA | NA | NA | 1,2 | NA |
| Califano | 2006 | 51 | 21 | 89 | 1,2,3,4 | NA |
| Kuroda | 2006 | 49 | 28 | 78 | 1,2,3 | NA |
| Ravazoula | 2006 | 37 | NA | 40 | 1 | 26 |
| Kim | 2005 | 48.8 | 28 | 83 | 1,2 | NA |
| Tangjitgamol | 2005 | 47 | NA | NA | 1,2,3 | NA |
| Chavez Blanco | 2004 | 41 | NA | NA | 1,2,3 | NA |
| Graflund | 2004 | 40 | 19 | 62 | 1,2,3 | 78 |
| Rosty | 2004 | 45 | 24 | 78 | 1,2,3,4 | 87 |
| Bellone | 2003 | NA | NA | NA | 1,2,4 | NA |
| Dellas | 2003 | NA | NA | NA | 1 | 100 |
| Heller | 2003 | 50 | 29 | 74 | 1,2,3 | NA |
| Niibe | 2003 | 60.2 | 39 | 81 | 3 | NA |
| Fuchs | 2002 | 50 | 24 | 89 | 1,2,3,4 | NA |
| Kedzia | 2002 | NA | NA | NA | 1 | 68 |
| Lee | 2002 | 51 | 20 | 85 | 1,2 | NA |
| Bhadauria | 2001 | 49 | NA | 70 | 2,3,4 | NA |
| Leung | 2001 | 50 | 46 | 65 | 1,2 | NA |
| Ngan | 2001 | 49 | 26 | 85 | 1,2 | 85 |
| Straughn | 2001 | 38 | 23 | 53 | 1,2,4 | NA |
| Chang | 1999 | NA | NA | NA | NA | NA |
| Kersemaekers | 1999 | 41 | 23 | 76 | 1,2 | 58 |
| Lakshmi | 1999 | NA | NA | NA | NA | NA |
| Mark | 1999 | NA | NA | NA | NA | NA |
| Nevin | 1999 | NA | NA | NA | 1,2 | NA |
| Nishioka | 1999 | NA | NA | NA | 1,2,3,4 | NA |
| Sharma | 1999 | 45 | 30 | 80 | 1,2,3 | NA |
| Mandai | 1997 | NA | NA | NA | 1,2 | NA |
| Ndubisi | 1997 | NA | NA | NA | 1,2,3,4 | NA |
| Kristensen | 1996 | NA | NA | NA | 1 | NA |
| Nakano | 1996 | NA | NA | NA | 1,2,3,4 | NA |
| Costa | 1995 | 51 | 19 | 87 | 1,2,3 | NA |
| Kihana | 1994 | NA | NA | NA | 1,2,3 | NA |
| Oka | 1994 | 61 | 25 | 83 | 3 | NA |
| Hale | 1992 | NA | NA | NA | 1,2,3 | NA |
| Berchuk | 1990 | NA | NA | NA | 1,2,3,4 | NA |

Abbreviations: NA = not available, FIGO = International Federation of Gynicology and Obstetrics, HPV = Human Papilloma Virus.

**B. Distribution of included studies by patients’ stages.**

| Stages | Number of studies | % Total | % Total Cum. |
| --- | --- | --- | --- |
| 1 | 5 | 8.9 | 8.9 |
| 1,2 | 11 | 19.6 | 28.6 |
| 1,2,3 | 12 | 21.4 | 50.0 |
| 1,2,3,4 | 21 | 37.5 | 87.5 |
| 1,2,4 | 2 | 3.6 | 91.1 |
| 2,3,4 | 3 | 5.4 | 96.4 |
| 3 | 2 | 3.6 | 100.0 |
|  | 0 | 0.0 | 100.0 |
| Total | 56 | 100.0 | 100.0 |
