# Supplementary material for: Prevalence of HER2 overexpression and amplification in cervical cancer: A systematic review and meta-analysis
Source: PLoS One. 2021 Sep 30;16(9):e0257976. doi: 10.1371/journal.pone.0257976 (PMC8483403; doi:10.1371/journal.pone.0257976)
Supplement: S10 File — (HTML) [file pone.0257976.s010.html]

Influence diagnostic


# Influence diagnostic

#### Boris Itkin

#### 2/6/2021

## R Markdown

---

# Identification of outliers

Two studies with absolute value of studentized residuals z-scores near 2.0 (Halle 2017 #7 and Gupta 2009 #18) have been identified as outliers.

---

Table. Studentized residuals.

```
##
##      resid     se       z
## 15  0.3196 0.1561  2.0474
## 5   0.2374 0.1274  1.8631
## 1   0.2420 0.1712  1.4134
## 12  0.2769 0.2042  1.3565
## 24  0.2769 0.2042  1.3565
## 19 -0.1885 0.1488 -1.2666
## 11 -0.2041 0.1629 -1.2523
## 17 -0.1923 0.1681 -1.1437
## 21  0.2476 0.2171  1.1402
## 16 -0.1587 0.1595 -0.9948
## 22 -0.1593 0.1839 -0.8662
## 23 -0.1546 0.1864 -0.8296
## 14 -0.1274 0.2023 -0.6297
## 6  -0.0990 0.1632 -0.6064
## 2  -0.1224 0.2054 -0.5959
## 3   0.0915 0.1791  0.5110
## 20 -0.0536 0.1764 -0.3040
## 13 -0.0440 0.1781 -0.2470
## 8   0.0398 0.1737  0.2289
## 18  0.0311 0.1689  0.1841
## 10 -0.0277 0.1667 -0.1659
## 7  -0.0202 0.1828 -0.1104
## 4  -0.0153 0.1731 -0.0881
## 9  -0.0154 0.1837 -0.0840
```

The same two studies were the biggest outliers in Baujat plot and Leave-one-out analysis plot (See below).

# Figure. Baujat plot

# Figure. Leave-one-out analysis

# Influence diagnostic

However, no influential study has been identified.

```
##
##    rstudent  dffits cook.d  cov.r tau2.del   QE.del    hat weight    dfbs inf
## 1    1.4134  0.3007 0.0885 1.0219   0.0216 169.8958 0.0424 4.2407  0.3007
## 2   -0.5959 -0.1061 0.0113 1.0393   0.0224 177.9967 0.0299 2.9876 -0.1060
## 3    0.5110  0.1022 0.0106 1.0571   0.0226 177.6260 0.0400 4.0029  0.1022
## 4   -0.0881 -0.0228 0.0005 1.0734   0.0229 178.6828 0.0435 4.3541 -0.0228
## 5    1.8631  0.5442 0.1968 0.7661   0.0145 107.6743 0.0528 5.2790  0.5221
## 6   -0.6064 -0.1482 0.0235 1.1164   0.0239 172.6503 0.0515 5.1516 -0.1490
## 7   -0.1104 -0.0249 0.0006 1.0589   0.0227 178.6767 0.0385 3.8462 -0.0248
## 8    0.2289  0.0450 0.0021 1.0702   0.0229 178.3807 0.0431 4.3113  0.0450
## 9   -0.0840 -0.0194 0.0004 1.0580   0.0227 178.6887 0.0380 3.8017 -0.0194
## 10  -0.1659 -0.0523 0.0031 1.1651   0.0252 178.3091 0.0520 5.1960 -0.0528
## 11  -1.2523 -0.2770 0.0754 1.0338   0.0217 168.2022 0.0473 4.7267 -0.2768
## 12   1.3565  0.2388 0.0567 1.0214   0.0219 174.6235 0.0299 2.9876  0.2391
## 13  -0.2470 -0.0540 0.0030 1.0636   0.0227 178.5173 0.0407 4.0705 -0.0540
## 14  -0.6297 -0.1138 0.0130 1.0402   0.0224 177.8807 0.0308 3.0844 -0.1137
## 15   2.0474  0.4749 0.2014 0.9456   0.0194 152.8017 0.0468 4.6774  0.4728
## 16  -0.9948 -0.2304 0.0534 1.0590   0.0223 166.2581 0.0506 5.0642 -0.2305
## 17  -1.1437 -0.2471 0.0608 1.0424   0.0221 172.0812 0.0448 4.4819 -0.2471
## 18   0.1841  0.0239 0.0007 1.2049   0.0262 176.9421 0.0528 5.2830  0.0242
## 19  -1.2666 -0.2906 0.0765 0.9746   0.0200 142.6677 0.0525 5.2534 -0.2880
## 20  -0.3040 -0.0665 0.0045 1.0655   0.0228 178.3900 0.0416 4.1611 -0.0665
## 21   1.1402  0.1881 0.0353 1.0243   0.0221 176.1403 0.0264 2.6438  0.1882
## 22  -0.8662 -0.1722 0.0298 1.0453   0.0223 176.5188 0.0375 3.7547 -0.1721
## 23  -0.8296 -0.1627 0.0266 1.0448   0.0224 176.8145 0.0365 3.6524 -0.1626
## 24   1.3565  0.2388 0.0567 1.0214   0.0219 174.6235 0.0299 2.9876  0.2391
```

Abbreviations: rstudent = studentized residuals, dffits = the change in the fitted values, cook.d = Cook’s distance, cov.r = covariance ratio, hat = leverage.

# Summary proportion and 95% confidence intervals of heterogeneity estimators after the removal of outliers

```
##      pred    ci.lb    ci.ub pi.lb   pi.ub cr.lb   cr.ub
##  0.049352 0.017138 0.093079     0 0.29811     0 0.29811
```

```
##
##        estimate   ci.lb   ci.ub
## tau^2    0.0269  0.0102  0.0514
## tau      0.1640  0.1011  0.2266
## I^2(%)  88.1313 73.8451 93.4099
## H^2      8.4255  3.8234 15.1744
```
