# Supplementary material for: Prevalence of HER2 overexpression and amplification in cervical cancer: A systematic review and meta-analysis
Source: PLoS One. 2021 Sep 30;16(9):e0257976. doi: 10.1371/journal.pone.0257976 (PMC8483403; doi:10.1371/journal.pone.0257976)
Supplement: S11 File — (DOCX) [file pone.0257976.s011.docx]

**S11 Supplementary file**

**Table 1. HER2 positivity prevalence in selected histological subtypes**

| Study | Year | ASCO/CAP compliance | HER2 method | Total N analyzed | Prevalence % |
| --- | --- | --- | --- | --- | --- |
| **Neuroendocrine** |  |  |  |  |  |
| Sukpan | 2011 | No | IHC | 100 | 2 |
| Tangjitgamol | 2005 | No | IHC | 24 | 0 |
| Straughn | 2001 | No | IHC | 16 | 0 |
| **Glassy cell** |  |  |  |  |  |
| Kuroda | 2006 | No | IHC | 11 | 45 |
| Kristensen | 1996 | No | IHC | 132 | 12 |
| **HPV-related adenocarcinoma, usual type** |  |  |  |  |  |
| Shi | 2020 | Yes | IHC, ISH | 111 | 10.8, 4.5 |
| **Mucinous adenocarcinoma, NOS** |  |  |  |  |  |
| Shi | 2020 | Yes | IHC, ISH | 20 | 15.0, 10.0 |
| Barbu | 2013 | Yes | IHC | 7 | 14.3 |
| **Gastric type adenocarcinoma** |  |  |  |  |  |
| Shi | 2020 | Yes | IHC, ISH | 34 | 14.7, 14.7 |
| Wong | 2020 | Yes | IHC | 4 | 25 |
| Nakamura | 2019 | Yes | IHC | 13 | 0 |
| Carleton | 2016 | Yes | IHC | 26 | 3.8 |
| **Clear cell adenocarcinoma** |  |  |  |  |  |
| Shi | 2020 | Yes | IHC | 4 | 25.0 |
| Ueno | 2013 | Yes | IHC | 13 | 23.5 |
| **Mesonephric** |  |  |  |  |  |
| Shi | 2020 | Yes | IHC | 1 | 0 |
| Khalimbekova | 2013 | Yes | IHC | 14 | 0 |
| **Serous adenocarcinoma and carcinomas with serous-like papillary and micropapillary components** |  |  |  |  |  |
| Shi | 2020 | Yes | IHC | 4 | 0 |
| Wong | 2020 | Yes | IHC | 14 | 21.4 |
| Barbu | 2013 | Yes | IHC | 2 | 50.0 |
| **Endometrioid adenocarcinoma** |  |  |  |  |  |
| Shi | 2020 | Yes | IHC | 3 | 0 |
| Barbu | 2013 | Yes | IHC | 4 | 25.0 |
| **Adenoid-cystic carcinoma** |  |  |  |  |  |
| Kim | 2005 | Yes | IHC | 1 | 100 |

Abbreviations: IHC = Immunohistochemistry, ISH = In Situ Hybridization, NOS = Not Otherwise Specified, ASCO/CAP = American Society of Clinical Oncology/American College of Pathologists.
