# Supplementary material for: Prevalence of HER2 overexpression and amplification in cervical cancer: A systematic review and meta-analysis
Source: PLoS One. 2021 Sep 30;16(9):e0257976. doi: 10.1371/journal.pone.0257976 (PMC8483403; doi:10.1371/journal.pone.0257976)
Supplement: S13 File — (DOCX) [file pone.0257976.s013.docx]

**S13 Supplementary file.**

**R code for meta-analysis of the prevalence of HER2 overexpression by ASCO/CAP compliance**

library(meta)

library(metafor)

library(readxl)

library(tidyverse)

dat=read_excel("C:\\Users\\HP\\OneDrive\\Cervix Her2\\Tercer metaanalisis\\X3_IHC all hisologies_con_patologo.xlsx")

nro <- dat %>%

select(ascocapcompl) %>%

filter(ascocapcompl == "compliant")

print(nro)

### MA transformacion Freeman Tukey

ies=escalc(xi=cases, ni=total, data=dat, measure="PFT", add=0)

print(ies)

### Pooling

pes.da=rma(yi, vi, data=ies, method="DL") # yi- tama?o de efecto individual=yi

# vi- varianza de cada estudio

# pes -pooled effect size

# DL -Dersimonian Laird Random

print(pes.da)

### Retrotransformacion

pes=predict(pes.da,transf=transf.ipft.hm, targ=list(ni=dat$total))

print(pes)

### Intervalos de confianza de los estimadores de heterogeneidad

ci.pes.da=confint(pes.da)

print(ci.pes.da)

### Creacion de Forrest plot

pes.summary=metaprop(cases, total, authoryear, data=dat, sm="PFT",

method.tau="DL", method.ci="NAsm")

forest(pes.summary,

xlim=c(0,100),

pscale=100,

rightcols=FALSE,

leftcols=c("studlab", "event", "n", "effect", "ci"),

leftlabs=c("Study", "Cases", "Total", "Prevalence", "95% C.I."),

xlab="Prevalence of HER2", smlab="Freeman-Tukey, DL, RE",

weight.study="random", squaresize=0.5, col.square="navy",

col.square.lines="navy",

col.diamond="maroon", col.diamond.lines="maroon",

pooled.totals=TRUE,

comb.fixed=FALSE,

fs.hetstat=10,

print.tau2=TRUE,

print.Q=TRUE,

print.pval.Q=TRUE,

print.I2=TRUE,

digits=1)

##### Estimadores de heterogeneidad y sus intervalos de confianza ###############

print(pes.da)

confint(pes.da, digits = 2)

#### Analisis de subgrupos

subganal.ascocapcompl=rma(yi, vi, data=ies, mods=asco_cap_compliance)

pes.da.1=rma(yi, vi, data=ies, mods=asco_cap_compliance=="0")

pes.da.0=rma(yi, vi, data=ies, mods=asco_cap_compliance=="1")

pes.subg.ascocapcompl=predict(subganal.ascocapcompl,

transf=transf.ipft.hm,

targ=list(ni=dat$total))

dat.samevar=data.frame(estimate=c((pes.da.1$b)[1], (pes.da.0$b)[1]),

stderror=c((pes.da.1$se)[1], (pes.da.0$se)[1]),

tau2=subganal.ascocapcompl$tau2)

pes.da.ascocapcompl=rma(estimate, sei=stderror,

method="DL",

data=dat.samevar)

pes.ascocapcompl=predict(pes.da.ascocapcompl, transf=transf.ipft.hm, targ=list(ni=dat$total))

print(pes.subg.ascocapcompl)#display subgroup 1 summary effect size

print(pes.subg.ascocapcompl) #display subgroup 2 summary effect size

print(subganal.ascocapcompl) #display subgroup analysis results

print(pes.ascocapcompl) #display recomputed summary effect size

###Forest plot de subgrupos moderador=ascocap

subganal.ascocapcompl=rma(yi, vi, data=ies, mods=~asco_cap_compliance, method="DL")

pes.summary=metaprop(cases, total, authoryear, data=dat, sm="PFT",

byvar=asco_cap_compliance,

tau.common=TRUE,

tau.preset=sqrt(subganal.ascocapcompl$tau2))

forest(pes.summary)

subganal.ascocapompl=rma(yi, vi, data=ies, mods=~asco_cap_compliance, method="DL")

pes.summary=metaprop(cases, total, authoryear, data=dat,

sm="PFT",

method.tau="DL",

method.ci="NAsm",

byvar=asco_cap_compliance,

tau.common=TRUE,

tau.preset=sqrt(subganal.ascocapcompl$tau2))

forest(pes.summary,

sortvar= - dat$year,

xlim=c(0,100),

pscale=100,

rightcols=FALSE,

leftcols=c("studlab","event","n","effect", "ci"),

leftlabs=c("Study","Positive","Total", "Proportion", "95% C.I."),

text.random="Pooled prevalence",

xlab="Prevalence of HER2", smlab="Freeman-Tukey, DL, ME",

weight.study="random", squaresize=0.5, col.square="navy",

col.diamond="maroon", col.diamond.lines="maroon",

pooled.totals=TRUE,

comb.fixed=FALSE,fs.hetstat=10,

print.tau2=TRUE,

print.Q=TRUE,

print.pval.Q=TRUE,

print.I2=TRUE,

digits=1,

print.subgroup.labels = TRUE,

bylab = c("ASCO/CAP compliant","ASCO/CAP non-compliant"),

print.byvar = TRUE)
