# Supplementary material for: Prevalence of HER2 overexpression and amplification in cervical cancer: A systematic review and meta-analysis
Source: PLoS One. 2021 Sep 30;16(9):e0257976. doi: 10.1371/journal.pone.0257976 (PMC8483403; doi:10.1371/journal.pone.0257976)
Supplement: Attachment — Submitted filename: Response to reviewers.docx [file pone.0257976.s014.docx]

Response to reviewers

Dear Reviewers

Thank you for your valuable comments. In the table below we are providing our answers to your questions.

Very best wishes.

The authors.

| **Reviewer 1** |  |
| --- | --- |
| Comment | Answer |
| 1. There are few grammar and phrasing errors, and professional editing is recommended | Thank you. We used editing services and we hope that now the manuscript is looking much better in this aspect. |
| 2. The term uterine cervical cancer should be replaced by cervical cancer in the title and throughout the manuscript | Thank you. All the terms ”uterine cervical cancer” were replaced by “cervical cancer” |
| 3.  3A  HER2 targeting has been proven to be an effective therapeutic approach in breast and uterine serous carcinoma. The clinical data in cervical cancer is limited. SUMMIT is a phase II basket trial that included cervical cancer patients and demonstrated evidence of activity.  3B  In this preliminary phase of drug development, comparisons to other disease sites that HER2 has an established role are needed.  3C  The authors mention heterogeneity in breast and uterine serous cancer, but they should present more specific data on the HER2 expression in breast and uterine serous carcinoma.  More information in the ‘Discussion’ is needed to discuss the methodology used and prevalence of overexpression and amplification in these disease sites | 3A  Thank you for this question. The SUMMIT Trial  ClinicalTrials.gov Identifier: NCT01953926.  To our knowledge, this trial is recruiting/recruited patients with solid tumors including CC with HER2 specific mutations, but not those with HER2 overexpression and/or amplifications.  We stated in the introduction section (lines 60-62) that HER2 specific mutations have been proven to be an effective therapeutic target (providing citations) but they are beyond the scope of our study as it is focused on HER2 amplification/overexpression which seldom (3.6% in breast cancer) coexists with activating mutations.(1) Similarly, in lung cancer Her2 amplification and activating mutation are distinct targets.  Following your suggestion, we added citation of the report on Neratinib efficacy in CC patients from SUMMIT trial to our overall bibliographic support, Ref 11. (2) Nonetheless, in this respect we think that a more detailed discussion of the SUMMIT trial results might be rather confusing than useful because, as we understand, the populations of HER2 mutated and HER2 amplified/overexpressed CC probably are two different ones with at most little overlap.(1, 3)  3B  Thank you for this very thoughtful suggestion. We added a comment on this providing a comparison of HER2 positivity rates in our study with to other disease sites where HER2 has an established role. Lines 390-397  3C  Thank you. We made this point of the discussion wider. We included the heterogeneity definition, quantitative data on HER heterogeneity prevalence and qualitative comparison between tumor types providing citation. Lines 433-440. |
| **Reviewer2** |  |
| 1.  Given that PLOS One public might not be expert on the oncology field, I would consider further contextualization of Cervical Cancer issues in some additional detail. For example:  1A  What is the disease burden worldwide that makes this study topic relevant?  1B  What implications would HER2 overexpression detection have either via IHC or FISH? What about diagnostic precision and accessibility in CC? | 1A  Thank you for this very important point. We introduced a paragraph about the disease burden and cervical cancer relevance for world’s public health. Lines 45-55  1B  Thank you for this interesting thought. A satisfactory for clinical use diagnostic precision (reproducibility) of HER2 testing by IHC and ISH is long established. We would prefer not to include the discussion about this, as primary studies in which our Review is based did not evaluate it. On the other hand, the ultimate criterion of the diagnostic accuracy (the power to separate those who will respond to anti-HER2 drugs from those who will not) of a HER2 testing by different methods (IHC, ISH, next generation sequencing), and their optimal arrangement in a sequence, is the clinical benefit of an anti-HER2 directed treatment. It can only be evaluated in clinical trials. Such studies in CC, to our knowledge, were not done yet. The accuracy may be context-dependent (we mean tissue- and drug-dependent). In this regard, we included comments in the discussion section. Lines 395-397 and 446-449  The main implication of a non-zero HER2 overexpression/amplification in CC would be a possibility to use HER2 as a target in clinical trials.  We stated in at the very end of the discussion section as a kind of conclusion that our data may be of practical aid in planning and organizing of such trials. |
| 2.  Human Papillomavirus has been clearly related as etiologic risk factor for developing Cervical Cancer. Usual approaches towards controlling or eliminating infection have shown efficacy for preventing CC, but this might have hindered research on other disease mechanisms in the past. Your approach is innovative as it highlights a hypothetical potential prognostic and/or predictive factor in CC, which may contribute as part of multimodal cancer treatment. It would be very interesting if the authors could comment on this relationship so that potential new research could address current knowledge gaps based on your findings (See Conesa-Zamora P et al. Exp Mol Pathol. 2013 Oct;95(2):151-5.) | Thank you for this suggestion. We added a comment on HPV role in CC (Lines 45-55).  Also, we added a comment on a possible connection between HPV related aneuploidy and the discordance between HER2 positivity rates determined by IHC and ISH observed in our study as it was suggested by Conesa-Zamora and co-workers. See below.  “As a possible explanation [of the heterogeneity], Conesa-Zamora et al. suggested that the increased copy number of chromosomes 17 is due to polyploidy, frequently present in advanced stages and HPV associated tumors ([23], Hu)”  Lines 406-409  Regarding the accessibility of some HER2 testing methods like ISH and NGS we are completely agreeing with you that it may be a problem in countries where CC is frequent. We added the comment “The accessibility of some HER2 testing methods in countries where CC is prevalent may be an issue.” Lines 452-452 of the revised Manuscript |

**New references appended to the Manuscript during its revision by authors**

2. World Health Organization. International Agency for Research on Cancer. Cancer Today.

103. Santin AD, Bellone S, Van Stedum S, Bushen W, Palmieri M, et al. (2005) Amplification of c‐erbB2 oncogene: a major prognostic indicator in uterine serous papillary carcinoma. Cancer 104: 1391-1397.

98. Richman SD, Southward K, Chambers P, Cross D, Barrett J, et al. (2016) HER2 overexpression and amplification as a potential therapeutic target in colorectal cancer: analysis of 3256 patients enrolled in the QUASAR, FOCUS and PICCOLO colorectal cancer trials. The Journal of pathology 238: 562-570.

11. Oaknin A, Friedman CF, Roman LD, D'Souza A, Brana I, et al. (2020) Neratinib in patients with HER2-mutant, metastatic cervical cancer: Findings from the phase 2 SUMMIT basket trial. Gynecol Oncol 159: 150-156.

100. Hu L, Potapova TA, Li S, Rankin S, Gorbsky GJ, et al. (2010) Expression of HPV16 E5 produces enlarged nuclei and polyploidy through endoreplication. Virology 405: 342-351.

3. Gupta SM, Mania-Pramanik J (2019) Molecular mechanisms in progression of HPV-associated cervical carcinogenesis. Journal of biomedical science 26: 28-28.

106. Dieci MV, Miglietta F, Griguolo G, Guarneri V (2020) Biomarkers for HER2-positive metastatic breast cancer: Beyond hormone receptors. Cancer Treat Rev 88: 102064.

105. Buza N (2021) HER2 Testing in Endometrial Serous Carcinoma: Time for Standardized Pathology Practice to Meet the Clinical Demand. Arch Pathol Lab Med 145: 687-691.

**References related to our response to Reviewers**

1.	Schettini F, Prat A. Dissecting the biological heterogeneity of HER2-positive breast cancer. The Breast. 2021 2021/08/05/.

2.	Oaknin A, Friedman CF, Roman LD, D'Souza A, Brana I, Bidard FC, et al. Neratinib in patients with HER2-mutant, metastatic cervical cancer: Findings from the phase 2 SUMMIT basket trial. Gynecol Oncol. 2020 Oct;159(1):150-6. PubMed PMID: 32723675. Pubmed Central PMCID: PMC8336424. Epub 2020/07/30. eng.

3.	ERBB2 cQ. Available from: https://[www.cbioportal.org/results?cancer_study_list=cesc_tcga_pan_can_atlas_2018&tab_index=tab_visualize&case_set_id=cesc_tcga_pan_can_atlas_2018_all&Action=Submit&gene_list=ERBB2](http://www.cbioportal.org/results?cancer_study_list=cesc_tcga_pan_can_atlas_2018&tab_index=tab_visualize&case_set_id=cesc_tcga_pan_can_atlas_2018_all&Action=Submit&gene_list=ERBB2).
